# Five-Day Treatment with *B. licheniformis* Along with Classical Vancomycin Treatment Was Effective in Preserving Gut Microbiota in Patients with *Clostridioides difficile* Infection

**DOI:** 10.3390/nu17040641

**Published:** 2025-02-11

**Authors:** Tae-Geun Gweon, Sang-Bum Kang, Soo-Young Na, Dong Jun Oh, Sang Wook Kim, Geom Seog Seo, Joo Young Cho

**Affiliations:** 1 Department of Internal Medicine, College of Medicine, The Catholic University of Korea, Seoul 02812, Republic of Korea; gweontae@naver.com (T.-G.G.); sangucsd@gmail.com (S.-B.K.); sktndud@hanmail.net (S.-Y.N.); 2 Department of Internal Medicine, Bucheon St. Mary’s Hospital, College of Medicine, The Catholic University of Korea, Bucheon 14662, Republic of Korea; 3 Department of Internal Medicine, Daejeon St. Mary’s Hospital, College of Medicine, The Catholic University of Korea, Daejeon 34943, Republic of Korea; 4 Department of Internal Medicine, Incheon St. Mary’s Hospital, College of Medicine, The Catholic University of Korea, Incheon 21431, Republic of Korea; 5 Department of Internal Medicine, Dongguk University Ilsan Hospital, Goyang 10326, Republic of Korea; mileo31@naver.com; 6 Department of Internal Medicine, Jeonbuk National University Hospital, Jeonju 54907, Republic of Korea; clickm@jbnu.ac.kr; 7 Department of Internal Medicine, Digestive Disease Research Institute, Wonkwang University Hospital, Iksan 54538, Republic of Korea; 8 Department of Internal Medicine, Cha Medical Center Gangnam, Seoul 06135, Republic of Korea

**Keywords:** *Clostridioides difficile* infection, *Bacillus licheniformis*, probiotic, microbiota

## Abstract

**Background/Objectives:** *Clostridioides difficile* infection (CDI) is an important nosocomial diarrheal disease. The benefits of the probiotic *Bacillus licheniformis* (*B. licheniformis*) in the preservation of intestinal microbiota have not been studied in patients with CDI to date. Therefore, we aimed to investigate the efficacy of *B. licheniformis* in preserving the intestinal microbiota in patients with CDI. **Methods:** A multicenter, randomized, placebo-controlled trial was carried out at six academic centers in Korea. Individuals diagnosed with mild to moderate CDI were included in this trial. CDI was treated with vancomycin 125 mg four times daily for two weeks. Along with vancomycin, *B. licheniformis* was administered for five days in this study, while a placebo was given to the placebo group. Microbiome analysis was performed before and five days after administering vancomycin and *B. licheniformis* or placebo, using 16S rRNA amplicon sequencing. Alpha and beta diversity was compared between the two groups. **Results:** A total of 35 participants were finally included in this study, with 16 in the study group and 19 in the placebo group. The alpha diversity was similar in both groups before CDI treatment. After five days of the administration of vancomycin and *B. licheniformis* or placebo, alpha diversity did not decrease in the study group (Chao1 index, *p* = 0.665; observed features, *p* = 0.692). In contrast, alpha diversity decreased in the placebo group (Chao1 index, *p* = 0.011; observed features, *p* = 0.011). Beta diversity did not differ between the two groups. **Conclusions:** The addition of *B. licheniformis* to vancomycin was effective in preserving gut microbiota in patients with CDI.

## 1. Introduction

*Clostridioides difficile* infection (CDI) is an important nosocomial diarrheal disease caused by the disruption of intestinal microbiota (dysbiosis) [1]. Antimicrobial use, chronic inflammation, and chronic diseases contribute to dysbiosis [2]. CDI is related to longer hospital stays and increased medical costs [3,4]. In the US, CDI is associated with roughly 500,000 infections, and mortality is reported at 30,000 deaths annually [5,6]. In the past, the incidence of CDI was lower in Asian countries compared to Western countries [7]. However, recent studies have shown that the incidence of CDI is increasing in Eastern countries [8,9].

CDI is regarded as a high-risk factor for post-infectious irritable bowel syndrome (IBS) [10], which is complicated in approximately 20% of patients with CDI [11]. The restoration of the disrupted intestinal microbiota is essential for recovery in patients with CDI [12]. Dysbiosis is related to CDI recurrence and the development of post-infectious IBS [13,14].

Fecal microbiota transplantation (FMT) is recommended as standard treatment for recurrent or refractory CDI [15,16,17,18]. Furthermore, dysbiosis can be restored after FMT [19]. Although various novel products have been developed for FMT to date [20], there is a potential for adverse events, including the transmission of pathogens [21].

Probiotics have emerged as an effective option for modulating human immunity [22] and can be used to restore the disrupted intestinal microbiota in various diseases. Probiotics have also been investigated for the treatment of CDI. A meta-analysis of randomized controlled trials (RCTs) reported that probiotic treatment effectively decolonizes *C. difficile* [23]. Another systematic review and meta-analysis showed a modest effect of probiotics for preventing recurrent CDI [24]. However, the number of patients that needed treatment was 42 in that review.

*Bacillus licheniformis* (*B. licheniformis*) is a Gram-positive spore-forming anaerobic bacterium. Spore-based probiotics have the ability to withstand acid in the stomach and can survive in the intestines [25,26]. The benefits of *B*. *licheniformis* have been investigated in various diseases, including colitis, infectious diarrhea, metabolic diseases, and cardiovascular disease [27]. In an animal study, *B*. *licheniformis* attenuated dextran sulfate sodium-induced colitis’s disrupted intestinal barrier integrity [28]. However, the efficacy of *B. licheniformis* in preserving the intestinal microbiota has not yet been studied in patients with CDI. It is important to identify new probiotics for the treatment of CDI.

In this multicenter trial, we investigated the efficacy of *B. licheniformis* on the intestinal microbiota using microbiome analysis in patients with CDI.

## 2. Methods

This was a multicenter, double-blinded RCT. Adults, aged ≥18 years, who were diagnosed with mild to moderate CDI were eligible for inclusion. CDI was diagnosed when the patients passed loose stools at least three times a day and tested positive for toxins [9]. The exclusion criteria were as follows: (1) recurrent CDI, (2) inflammatory bowel disease, (3) a history of immunosuppressive agent use or chemotherapy, (4) the use of probiotics three months prior to inclusion, and (5) inability to discontinue causative antibiotics for CDI at the time of inclusion. A toxin assay was performed using a Wampole^TM^ C. DIFF QUIK CHEK COMPLETE^TM^ (TechLab, Blacksburg, VA, USA).

After obtaining informed consent, the study participants were randomized into the study and placebo groups. *B. licheniformis* 250 mg or placebo three times per day was administered for five days in the study and placebo groups, respectively. The placebo, which contained starch as its main component, was designed to match the study drug in color and shape. CDI treatment was performed using 125 mg of vancomycin four times per day for both groups. Enrollment was performed between July 2021 and December 2022 at six academic hospitals in Korea: Bucheon St. Mary’s Hospital, Incheon St. Mary’s Hospital, Daejeon St. Mary’s Hospital, Wonkwang University Hospital, Jeonbuk National University Hospital, and Dongguk University Ilsan Hospital. The institutional review boards of the participating hospitals approved the study protocol.

### 2.1. Microbiome Analysis

Microbiome analysis was performed before and five days after the administration of vancomycin and *B. licheniformis* or placebo. The stool was collected from the study participants, and at least 1 g was stored in a THERAbiome stool kit (THERAGEN Health, Seongnam, Republic of Korea). Using the sampling kit, the stool microbiome was preserved at room temperature for up to 65 days after collection [29]. The stool kit was delivered to each hospital within three days of collection. The participating hospitals then sent the stool kits to Theragen Bio (Seongnam, Republic of Korea) every week.

The protocol for microbiome analysis was described previously [29,30]. Metagenomic DNA was extracted from stool samples using QIAmp (Qiagen, Germany). Long-read metagenomic sequencing was performed using MiSeq software (version 4.0) (Illumina, CA, USA). The V3–V4 region of microbial 16S rRNA genes was targeted.

Alpha diversity, beta diversity, and microbial species were compared between the two groups using 16S rRNA amplicon sequencing [30]. Alpha diversity was compared using the Shannon, Simpson, and Chao 1 indices and the observed features. Beta diversity was investigated using UniFrac analysis. Bacterial 16S rRNA sequencing data were analyzed using the QIIME2 bioinformatics pipeline (http://qiime2.org, accessed on 26 September 2022).

The primary endpoint of this study was microbial changes after five days of treatment. Patient characteristics, stool frequency, and form measured using the Bristol stool scale (BSS); CDI treatment results; and adverse events during the trial were investigated. The participants were followed up for two months after inclusion.

### 2.2. Statistical Analysis

This was a pilot study. We planned to include 40 patients with CDI (20 patients per group). The alpha and beta diversities of the two groups were compared using the Kruskal–Wallis test. Continuous data were presented as means or medians, as appropriate for clinical data. The Mann–Whitney U-test was used for continuous variables. The chi-square and Fisher’s exact tests were used for binary outcomes, as appropriate. Statistical analyses were performed using R software (version 4.3).

## 3. Results

The 40 participants were randomized in a 1:1 ratio. After excluding 5 participants (withdrawal of informed consent, *n* = 2; improper collection of stool samples, *n* = 3), 35 participants (study group, *n* = 16; placebo, *n* = 19) were finally included. The demographic characteristics of the study participants are presented in Table 1. Age, sex, body mass index, admission status, duration of antibiotic use, stool form, and stool frequency were comparable between the two groups.

### 3.1. Microbiome Analysis at Randomization

**Figure 1:**
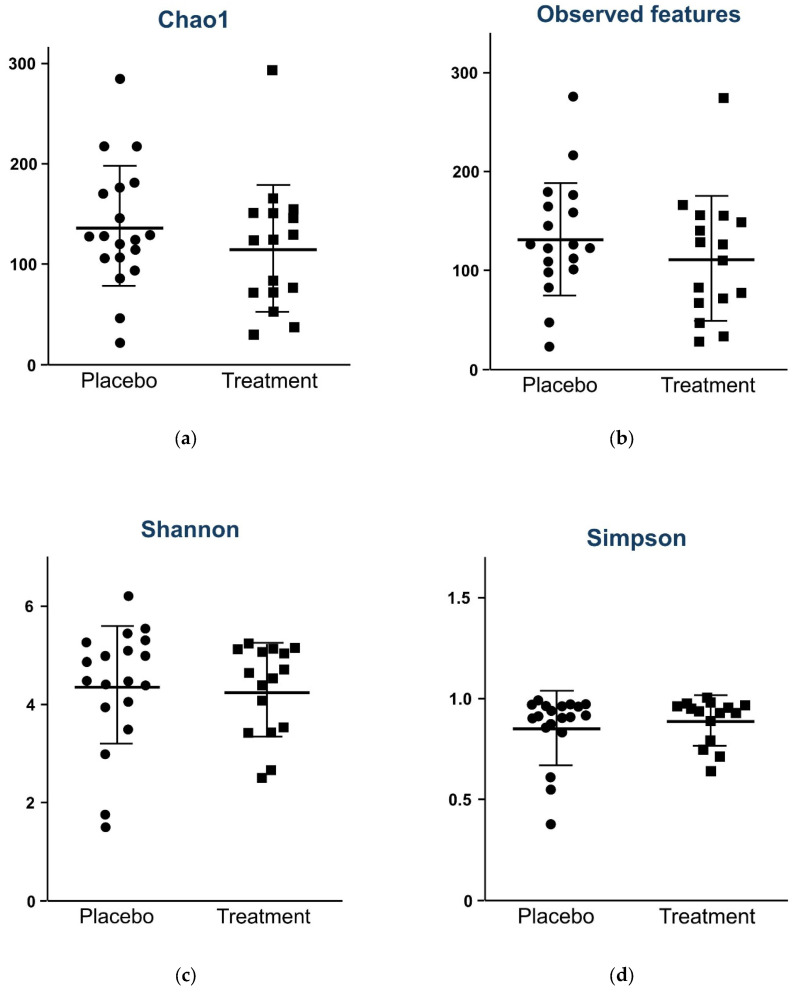
Comparison of alpha diversity between probiotic and placebo groups at randomization. Alpha diversities measured using Chao1 index, *p =* 0.337 (**a**); observed features, *p =* 0.290 (**b**); Shannon diversity index, *p =* 0.832 (**c**); and Simpson diversity index, *p =* 0.806 (**d**), are comparable between the two groups.

The alpha diversity measured at randomization was comparable between the two groups (Chao 1 index, *p* = 0.337; observed features, *p* = 0.290; Shannon index, *p* = 0.832; Simpson index, *p* = 0.806; Figure 1).

### 3.2. Microbiome Analysis at Five Days After Treatment

**Figure 2:**
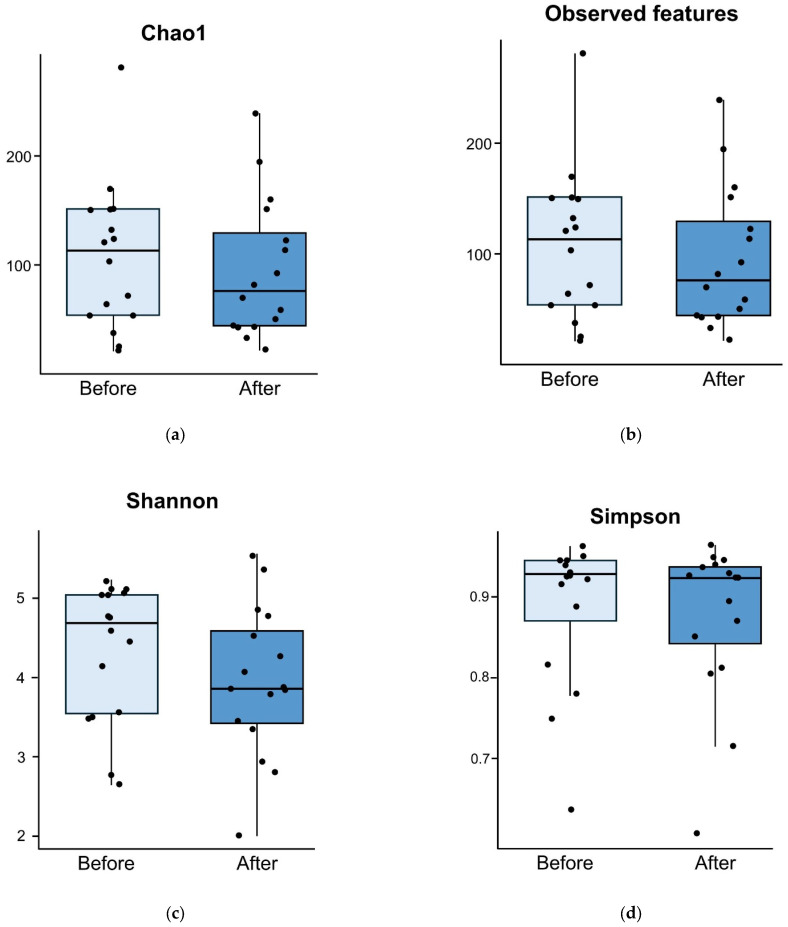
Comparison of alpha diversity for probiotic group between before and five days after treatment. Alpha diversities measured using Chao1 index (*p =* 0.665) (**a**), observed features (*p* = 0.692) (**b**), Shannon diversity index (*p* = 0.287) (**c**), and Simpson diversity index (*p* = 0.806) (**d**) are comparable before and after treatment.

The results of the microbiome analysis five days after treatment are presented in Figure 2. In the study group, alpha diversity did not decrease after five days of the administration of the study drug and vancomycin (Chao1 index, *p =* 0.665; observed features, *p* = 0.692; Shannon index, *p* = 0.287; Simpson index, *p* = 0.361; Figure 2).

**Figure 3:**
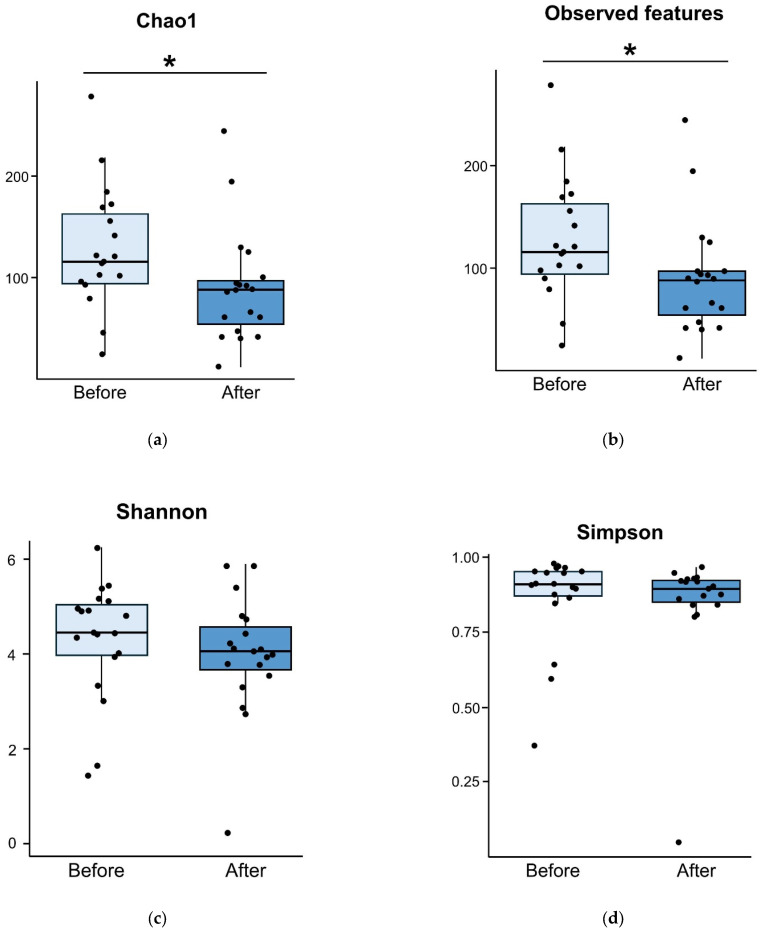
Comparison of alpha diversity for placebo group between before and five days after treatment. Alpha diversity measured using Chao1 index (*p* = 0.011) (**a**) and observed features (*p* = 0.011) (**b**) are decreased five days after treatment. However, alpha diversities measured using Shannon (*p* = 0.138) (**c**) and Simpson (*p* = 0.311) (**d**) diversity indices are comparable. * Statistically significant.

However, in the placebo group, alpha diversity significantly decreased five days after treatment (Chao1 index, *p* = 0.011; observed features, *p* = 0.011; Figure 3a,b). The alpha diversity measured using the Shannon (*p* = 0.138, Figure 3c) and Simpson (*p* = 0.311, Figure 3d) indices was similar before and after treatment in the placebo group.

**Figure 4:**
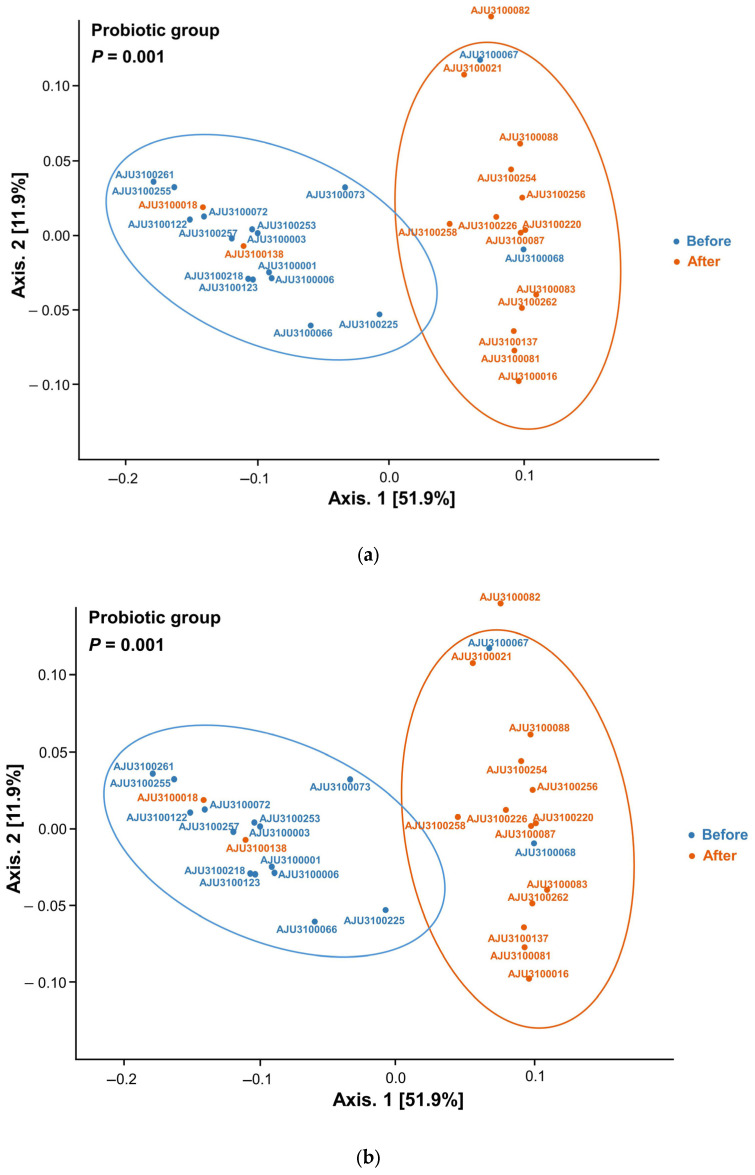

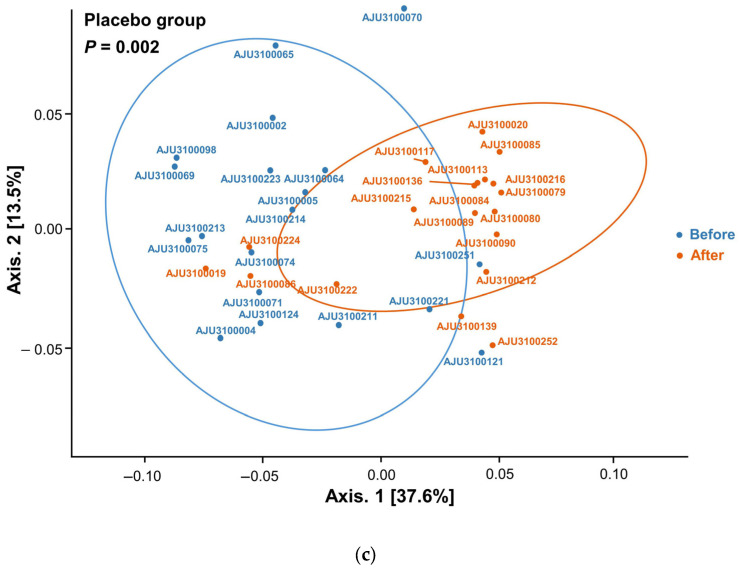
Comparison of beta diversity. Beta diversity differs between before and five days after treatment in probiotic group (*p =* 0.001) (**a**). Beta diversity differs between before and five days after treatment in placebo group (*p =* 0.002) (**b**). Beta diversity is comparable between probiotic and placebo groups (**c**).

Beta diversity after treatment, measured using weighted UniFrac, differed from that before treatment in the study (*p =* 0.001, Figure 4a) and placebo (*p =* 0.002, Figure 4b) groups. However, beta diversity did not differ between the placebo and study groups (Figure 4c).

### 3.3. Treatment Results

All patients were cured after two weeks of vancomycin treatment. No adverse events occurred during treatment. Stool form and the frequency of defecation did not differ between the two groups. Five days after treatment, 65.7% and 51.4% of participants showed an improvement in stool type (from BSS type 6 and 7 to type 3–5) and frequency (from ≥3/day to 1–2/day), respectively (Table 2).

## 4. Discussion

In this pilot study, a combination of the probiotic *B. licheniformis* and vancomycin showed positive efficacy in preserving the intestinal microbiota in patients with CDI during the early stages of this disease. In the placebo group, alpha diversity was measured using the Chao1 index, and the observed features decreased after five days of treatment. However, the alpha diversity measured using all diversity indices did not decrease in the probiotic group.

The benefits of probiotics have been investigated in clinical practice, where the temporal disruption of the intestinal microbiota was implicated, such as *Helicobacter pylori* (*H. pylori*) eradication or bowel preparation. The addition of a probiotic supplement was effective in restoring beneficial microbiota during *H. pylori* eradication using bismuth quadruple therapy [31]. In another study, the efficacy of probiotic treatment during bowel preparation was investigated. Probiotics have shown positive effects on preserving intestinal microbiota and decreasing mild abdominal pain after bowel preparation [32].

The use of probiotics in patients with CDI is still controversial [33]. The American Gastroenterological Association guidelines suggest only four types of probiotics for preventing CDI in patients receiving antibiotic treatment [34]. In this study, *B. licheniformis* was used as a supplement for CDI treatment because this strain has not been investigated to date for the treatment of CDI. In our study, we excluded those who could not cease causative antibiotics for CDI at the time of inclusion because the continuous use of antibiotics is related to the deleterious effects of probiotics. Although the cessation of causative antibiotics is recommended in guidelines [15,35], it is challenging to cease them in cases where the index infection is not controlled. To date, the proper duration of probiotic treatment has not been determined. We hypothesized that dysbiosis was the most severe at the time of CDI diagnosis due to recent antibiotic treatment and diarrhea. Thus, in this study, probiotic *B. licheniformis* was prescribed for five days in the early stages of CDI. Although stool frequency and form improved five days after vancomycin treatment, alpha diversity in the placebo group decreased compared to the baseline at randomization, as measured using the Chao1 index and observed features representing microbial richness. However, the evenness of microbial diversity measured using the Shannon and Simpson indices did not differ before treatment and five days after treatment in both groups. The *B. licheniformis* probiotic was partly effective in preserving microbial richness during the early stages of CDI. In this study, beta diversity differed before and after the administration of the probiotic and placebo, along with vancomycin, for the two groups. However, beta diversity did not differ between the two groups after completing the *B. licheniformis* probiotic and placebo. This finding suggests that the overall structure of the microbial community in the probiotic group was similar to that of the placebo group. The addition of a single strain did not affect the compositional change in the microbial structure.

The recurrence rate of CDI is estimated to be approximately 20% [36]. Dysbiosis is more severe in patients with recurrent CDI than in those with primary CDI [12]. Therefore, the preservation of the intestinal microbiota is important for treating CDI. It is acknowledged that maintaining microbial diversity is associated with preventing recurrence. The mechanisms underlying the efficacy of probiotics in treating CDI have also been investigated. One study reported that the engineered probiotic *E. coli* Nissel 1917 limited the germination of endospores of *C. difficile* and thereby the growth of *C. difficile* [37]. Another study suggested that the probiotic *Bacillus amyloliquefaciens* showed an anti-CDI effect by synthesizing extracellular antimicrobial compounds [38]. However, these studies were performed using animal models. The health benefits of the *B. licheniformis* probiotic have been linked to the modulation of the intestinal microbiota, anti-inflammatory activity, and improvement in the lipid profile. However, these effects have not yet been investigated in patients with CDI.

This study has several limitations. First, although CDI treatment is recommended for 10–14 days, microbial analysis was not performed at the time of treatment completion. We investigated the effects of probiotics in the early stages of treatment (five days after treatment). Second, a long-term follow-up was not performed. Consequently, it remains unclear whether the beneficial effects of probiotic supplementation observed five days post-CDI treatment persist over an extended period. Third, the mechanism of action of *B. licheniformis* in patients with CDI was not investigated. Fourth, the sample size was small and was not calculated. Because this was a pilot study, the sample size could not be calculated. Fifth, a detailed analysis investigating the specific taxa affected by probiotic was not performed.

## 5. Conclusions

In conclusion, the addition of *B. licheniformis* helped preserve microbial richness in patients with CDI during the early stages of treatment. However, our study has several limitations, including a small sample size and short-term follow-up. Future studies are needed to investigate the long-term effects of probiotic treatment and to reveal the mechanisms of probiotic treatment in patients with CDI. The effects of this strain on CDI recurrence should be investigated in future large-scale studies.

## Figures and Tables

**Table 1:** Baseline characteristics.

Characteristics	Probiotic (*n* = 16)	Placebo (*n* = 19)	*p*
Age, median (IQR)	70.5 (60.5, 78.0)	62.0 (54.5, 72.0)	0.226
Male, *n* (%)	7 (43.8%)	5 (26.3%)	0.468
Body mass index, median (IQR)	23.3 (19.3, 25.3)	23.1 (20.6, 25.8)	0.441
Inpatient, *n* (%)	14 (87.5%)	17 (89.5%)	1.000
Duration of antibiotic treatment, *n* (%)			1.000
≥2 weeks	4 (25.0%)	5 (26.3%)	
<2 weeks	12 (75.0%)	14 (73.7%)	
Smoking, *n* (%)			0.677
Current or ex-smoker	4 (25.0%)	3 (15.8%)	
Non-smoker	12 (75.0%)	16 (84.2%)	
Alcohol use, *n* (%)			0.379
Yes	4 (25.0%)	2 (10.5%)	
<1/month	12 (75.0%)	17 (89.5%)	
BSS, *n* (%)			1.000
Type 6	9 (56.2%)	10 (52.6%)	
Type 7	7 (43.8%)	9 (47.4%)	
Stool frequency, number/day (%)			0.152
3	13 (81.2%)	10 (52.6%)	
≥4	3 (18.8%)	9 (47.4%)	

IQR, interquartile range; BSS, Bristol stool scale.

**Table 2:** Stool characteristics at 5 days after treatment.

Characteristics	Probiotic (*n* = 16)	Placebo (*n* = 19)	*p*
BSS, *n* (%)			0.713
Type 3, 4, 5	10 (62.5)	13 (68.4)	
Type 6, 7	6 (37.5)	6 (31.6)	
Stool frequency, *n* (%)			0.947
1–2/day	8 (50.0%)	10 (52.6%)	
3–4/day	5 (31.2%)	5 (26.3%)	
≥5/day	3 (18.8%)	4 (21.1%)	

BSS, Bristol stool scale

## Data Availability

The data presented in this study are available on request from the corresponding author due to privacy policies.

## Abbreviations

The following abbreviations are used in this manuscript:

CDI	*Clostridioides difficile* infection
IBS	Irritable bowel syndrome
FMT	Fecal microbiota transplantation
RCT	Randomized controlled trial
BSS	Bristol stool scale
